# LEAPS Vaccine Incorporating HER-2/neu Epitope Elicits Protection That Prevents and Limits Tumor Growth and Spread of Breast Cancer in a Mouse Model

**DOI:** 10.1155/2017/3613505

**Published:** 2017-03-26

**Authors:** Ken S. Rosenthal, Sarah Stone, Gary Koski, Daniel H. Zimmerman

**Affiliations:** ^1^College of Medicine, Roseman University of Health Sciences, 10530 Discovery Drive, Las Vegas, NV 89135, USA; ^2^Northeast Ohio Medical University, Rootstown, OH 44272, USA; ^3^Kent State University, Kent, OH 44240, USA; ^4^CEL-SCI Corporation, 8229 Boone Blvd, Suite 802, Vienna, VA 22182, USA

## Abstract

The prototype J-LEAPS T cell vaccine for HER-2/neu breast cancer (J-HER) consists of the murine HER-2/neu_66–74_ H-2^d^ CD8 T cell epitope covalently attached through a triglycine linker to the J-immune cell binding ligand (ICBL) (human *β*2 microglobulin_38–50_ peptide). The J-ICBL was chosen for its potential to promote Th1/Tc1 responses. In this proof-of-concept study, the ability of J-HER to prevent or treat cancer was tested in the TUBO cell-challenged BALB/c mouse model for HER-2/neu-expressing tumors. The J-HER vaccine was administered as an emulsion in Montanide ISA-51 without the need for a more potent adjuvant. When administered as a prophylactic vaccination before tumor challenge, J-HER protected against tumor development for at least 48 days. Despite eliciting protection, antibody production in J-HER-immunized, TUBO-challenged mice was less than that in unimmunized mice. More importantly, therapeutic administration of J-HER one week after challenge with TUBO breast cancer cells limited the spread of the tumors and the morbidity and the mortality in the challenged mice. The ability to elicit responses that prevent spread of the TUBO tumor by J-HER suggests its utility as a neoimmunoadjuvant therapy to surgery. Individual or mixtures of J-LEAPS vaccines can be readily prepared to include different CD8 T cell epitopes to optimize tumor therapy and customize treatment for individuals with different HLA types.

## 1. Introduction

CD8 T cells optimized for antitumor activity are initiated by interleukin 12- (IL12-) producing dendritic cells (DC1) presenting peptide epitopes from tumor proteins on MHC I antigens [1]. IL12 promotes production of CD4 T cells (Th1) and CD8 T cells (Tc1) which produce interferon *γ* (IFN*γ*) [1–4] and promotes the production of CD8 cytotoxic T cells (CTL) that are more sensitive to antigen than T cells induced by other responses [1, 5–7]. Th1/Tc1 responses are important for generating antitumor protections [1, 4], and IL12 also promotes other supportive antitumor activities [8].

The Ligand Epitope Antigen Presentation System (LEAPS) technology converts a small peptide containing a disease-specific epitope into an immunogen while simultaneously directing the nature of the subsequent immune response by covalently attaching the peptide to an immune cell binding ligand (ICBL) through a triglycine linker [9]. The J-ICBL is a peptide from the beta-2-microglobulin component of MHC I ((aa38–50) (DLLKNGERIEKVE) [10]) and promotes Th1/Tc1-type responses [11–16] to MHC I-binding peptides as small as a minimal CD8 T cell epitope of 8 amino acids. The G- or derG-ICBL is a peptide from the beta chain of human MHC II ((aa135–149) (DGQEEKAGVVSTGLI)) and promotes Th2-type responses [11, 17].

J-LEAPS vaccines have elicited immune responses to *Mycobacteria tuberculosis* antigens [11] and HIV [18], initiated antimicrobial prophylaxis to HSV-1 [12–15] and influenza [16], and provided an immunomodulating influence on ongoing autoimmune responses [19–21], as demonstrated in appropriate mouse models. Protection from lethal challenge with HSV-1 followed immunization with JH1 [14], an 8-amino acid CD8 T cell epitope from the HSV-1 ICP27 protein whereas the component peptides elicited no protection. An antibody to this intracellular protein was not produced and would not have been protective [22]. Immunization with a conjugate containing a longer peptide from HSV-1 glycoprotein D, JgD, elicited protection from disease and death that was dependent upon IFN*γ*, CD8, and CD4 cells, as indicated by ablation studies, but only generated a Th1/Tc1-associated antibody response (IgG2a/IgG1 > 1) in those mice with minor breakthrough disease [15]. The GgD vaccine elicited Th2-associated antibody responses, but neither the GH1 [14] nor the GgD [15] vaccines elicited protections in the HSV-1-challenge models. J-LEAPS vaccines activate and direct the nature of the subsequent immune response to a Th1/Tc1 response by promoting the maturation of mouse and human precursor cells into IL12-producing dendritic cells (DC1s) which also present the antigenic peptide in the conjugate to CD8 T cells [12, 13, 16]. Based on these previous findings, we hypothesized that a heteroconjugate vaccine combining the LEAPS J-ICBL with a CD8 T cell epitope from breast cancer cells would elicit potent antitumor responses.

Cells from breast cancer and a variety of other human malignancies express the HER-2/neu cell surface receptor and are dependent upon its function [23]. Peptides from HER-2/neu are presented on MHC I as antigens that can be recognized by CD8 T cells, and several of the peptides have been tested as antitumor vaccines in mouse models [24] and considered for humans [25]. The peptide vaccines administered with incomplete Freund's adjuvant (IFA) are ineffective but, when administered with powerful adjuvants such as a toll-like receptor agonist, elicit antitumor CD8 T cell responses [24].

In this proof-of-concept study, a LEAPS vaccine for HER-2/neu-positive breast cancer was prepared by covalent attachment of the J-ICBL using a triglycine spacer to a prominent CTL-eliciting minimal CD8 T cell epitope from HER-2/neu (aa66-74: TYVPANASL) for BALB/c mice (J-HER) [24]. The vaccine, administered as an emulsion with an IFA-like Montanide ISA-51 oil in water emulsion adjuvant, was tested for its ability to elicit prophylactic and therapeutic responses in mice challenged with TUBO cells. TUBO cells [26] were originally obtained from a spontaneous mammary gland tumor that arose in a BALB-neuT mouse and generate HER-2/neu-expressing tumors.

## 2. Materials and Methods

### 2.1. Vaccine

J-HER peptide (DLLKNGERIEKVE-GGG-TYVPANASL, mwt 2631) was synthesized by FMOC chemistry, purified ≥95% by RP-HPLC, mass-determined by MS to ±2 atm, lyophilized by 21st Century Biochemicals, Marlboro, MA, and supplied as an acetate salt. The peptide was resuspended in sterile Hank's balanced salt solution (HBSS) to 2 mM after checking pH and adjusting if necessary to 7.4. Prior to immunization, a 1 : 1 emulsion of J-HER was prepared in Montanide ISA-51 (SEPPIC Inc., Fairfield, New Jersey) adjuvant.

### 2.2. TUBO Cell Tissue Culture

TUBO cells [26] were kind gifts of Dr. Guido Forni (University of Turin). The cells were grown in tissue culture in RPMI 1640 medium supplemented with 10% fetal bovine serum (HyClone), penicillin (100 IU/mL), streptomycin (100 g/mL), and 2.25 mM NaHCO3 at 37°C. TUBO cells were demonstrated to be free of mycoplasma and contaminating viral infections and were limited to low passage for implantation in BALB/c female mice (Jackson Laboratories, Bar Harbor, ME). Mice were 7–10 weeks old when implanted with TUBO cells.

### 2.3. Tumor Challenge

TUBO cells were injected subcutaneously in a 50–100 *μ*L volume into two parallel sites on the abdomen. A tumor cell amount of 2.5 × 10^5^ per site was determined to be a lethal challenge. Mice were examined every other day. Tumor volume was determined by measuring along the longest axis of the tumor, along the second line perpendicular to this axis, and along the height and are presented as an average for the survivors of the group. Mice were also evaluated for other visible signs indicating poor health, including poor hygiene, lethargy, coat matting, ulceration, wasting and belly swelling due to ascites, inability to reach food and water, or excessive tumor size (>50% body mass). When necessary, mice were euthanized with carbon dioxide. All animal studies were reviewed and approved by the Institutional Animal Care and Use Committee (IACUC) at Northeast Ohio Medical University.

### 2.4. Prophylaxis Challenge Model

Mice were immunized in three locations, bilaterally into the abdomen at 40 *μ*L per site followed by a 20 *μ*L injection into the dorsal nape of the neck with an emulsion of HBSS as the control (*n* = 14) or J-HER peptide ((DLLKNGERIEKVE-GGG-TYVPANASL) in HBSS (1 mM (0.26 mg total peptide per immunization)) (*n* = 7) in Montanide ISA-51 adjuvant. Immunizations were at 3 weeks and 1 week prior to challenge. TUBO cell challenge was administered by subcutaneous implantation of 2.5 × 10^5^ cells into a site on either side of the lower abdomen.

### 2.5. Therapeutic Challenge Model

Tumor development was initiated by subcutaneous injection of TUBO cells into two sites (2.5 × 10^5^ cells per site) in mice. Mice were treated with the J-HER vaccine (*n* = 9) or the control (*n* = 9), as described for the prophylaxis trial, one week after initiation of the challenge and repeated every two weeks after the initial treatment. In addition to tumor volume and mortality, a disease score for each mouse was calculated by the following formula: tumor load (1–3 points), presence of skin necrosis at the site of the tumor (+1 point), ascites (+1 point), general unkempt/unhealthy appearance (+1 point), and death (+4 points); and presented as an average for the group.

### 2.6. ELISA for IgG Antibody to HER-2/neu

Sera were obtained following blood collection after a lancet puncture of the submandibular vein (lancets from Goldenrod, 4 mm). Blood was permitted to clot at room temperature for 2–3 hours and then an additional 2–3 hours at 4°C, centrifuged to separate clot from serum and serum withdrawn, and placed in labeled storage vials. Sera were frozen until analysis.

ELISA was used to test for the presence of IgG antibody reactive to the extracellular domain (ECD) of recombinant HER-2/neu. EIA/RIA plates were coated with 50 *μ*L of a recombinant HER-2/neu ECD (ACROBiosystems, Newark, DE) at a concentration of 5 *μ*g/mL in 0.15 M sodium carbonate bicarbonate buffer pH 9.6 and incubated overnight at 4°C. The plates were then washed three times with PBS/Tween (0.05%) and then blocked with 200 *μ*L of 1% bovine serum albumin (BSA) in PBS/Tween for 1 hour. The plates were washed again three times using the same wash buffer, and 50 *μ*L of sera was added at a dilution of 1 : 10 or 1 : 100 in 1% BSA. The plates were incubated at room temperature for 2 hours, then washed three times. The presence of bound HER-2 antibodies in each well was detected by adding 50 *μ*L of antimouse IgG secondary antibody conjugated to HRP (Thermo Scientific) for 1 hour, washing three times, developing the plate with 50 *μ*L/well of TMB (3,3′,5,5′-tetramethylbenzidine) substrate (KPL, Kirkegaard & Perry Laboratories Inc., Rockville, MD), stopping the reaction with 25 *μ*L/well of 1 N HCl, and reading the optical density of the resulting reaction at 450 nm.

### 2.7. Statistics

GraphPad Prism 6 Software (GraphPad, La Jolla, CA) was used to plot the data and also for recalculation of statistics. The two-way ANOVA Fisher LSD test was applied at 95% confidence interval to determine the statistical significance between the groups. A value of *p* < 0.05 is considered significant.

## 3. Results

### 3.1. J-HER Immunization to Prevent Tumor Development

Mice were immunized with an emulsion of HBSS or J-HER peptide (DLLKNGERIEKVE-GGG-TYVPANASL) (2 mM) in Montanide ISA-51 adjuvant (100 microliters per mouse) at 3 weeks and 1 week prior to challenge with subcutaneous implantation of 2.5 × 10^5^ TUBO cells into two sites on either side of the lower abdomen. J-HER includes a minimal MHC I-binding CD8 T cell epitope of nine amino acids from HER2/neu (peptide p66–74, TYVPANASL) covalently attached to the J-ICBL through a triglycine linkage.

**Figure 1:**
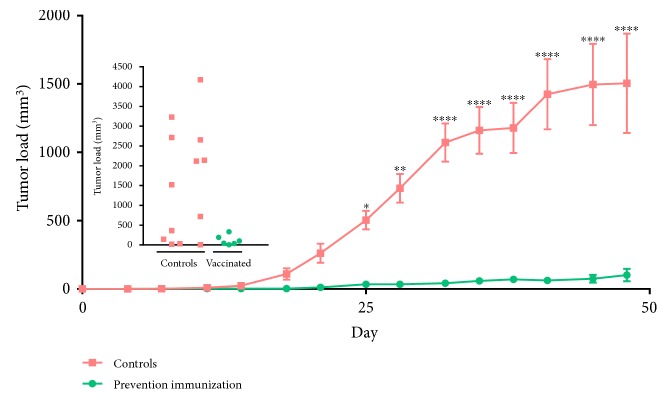
J-HER prevention of TUBO cell tumor development. Mice were immunized with HBSS in Montanide ISA-51 adjuvant (orange rectangles) or J-HER in adjuvant (green circles), which was repeated 14 days later, and one week later, immunized mice (*n* = 7) and unimmunized mice (*n* = 14) were challenged with 2.5 × 10 [7] TUBO cells in each of two sites. Tumor volume is presented with respect to day after tumor challenge. Inset shows total tumor size for individual mice at day 48, end of experiment. (^∗^*p* < 0.05; ^∗∗^*p* < 0.01; ^∗∗∗^*p* < 0.001; ^∗∗∗∗^*p* < 0.0001.)

**Figure 2:**
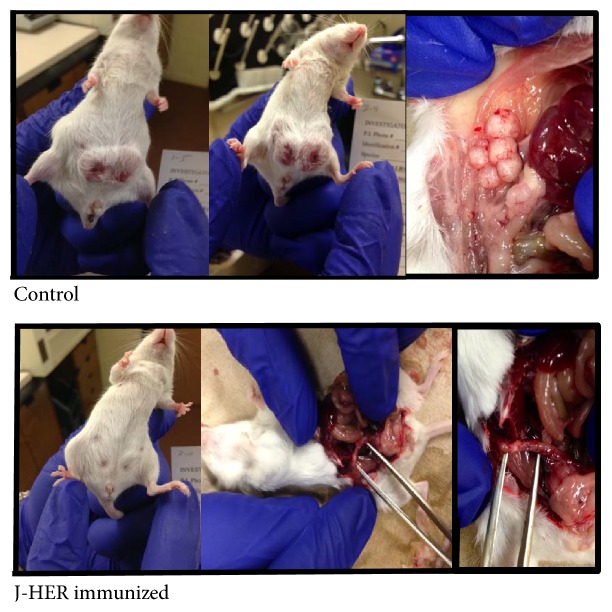
Appearance of representative mice from the J-HER prevention trial described in Figure 1. TUBO cell tumor development is shown for the control and J-HER-immunized mice on day 49 after tumor challenge.

Unimmunized mice developed measureable tumors within 14 days after injection of TUBO cells into the abdomen (Figure 1). Although the TUBO cells were injected subcutaneously, tumor growth extended into the abdomen and through the skin (Figure 2). In addition to the tumor swelling at the two implantation sites, the control mice had ruffled fur on the abdomen and exhibited poor hygiene, lethargy, and matted coats. Necropsy of representative animals showed multiple nodules within the mesentery of the abdomen with visible vascularization of the nodules (see Figure 2). In contrast, for the J-HER-immunized mice, there was minimal evidence of tumor development or disease signs over the course of the trial, 48 days after injection of TUBO cells. The bellies of the J-HER-vaccinated mice were flat, the fur was smooth, and on necropsy, vaccinated mice did not show the presence of tumors.

**Figure 3:**
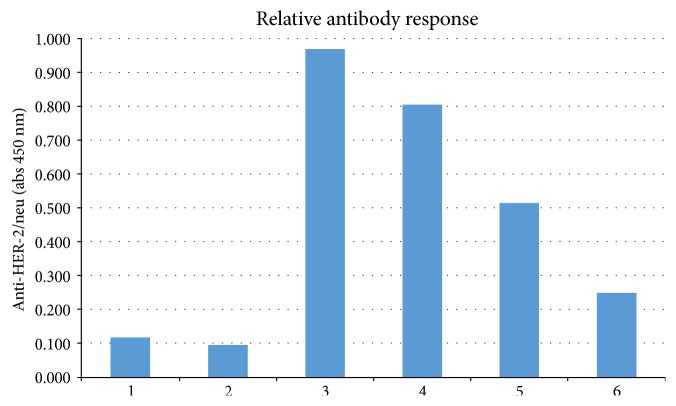
Anti-HER-2/neu antibody levels. Serum obtained from mice from the experiment shown in Figure 1 on day 7 after tumor challenge (collected on day 28 after the first vaccination) was evaluated by ELISA at two serum dilutions for antibody to HER-2/neu. Mice that were unimmunized and unchallenged provided the control serum (“nontumor”). Nontumor: 1 (1 : 10), 2 (1 : 100); tumor only: 3 (1 : 10), 4 (1 : 100); tumor + vaccine: 5 (1 : 10), 6 (1 : 100).

Serum was obtained from mice on day 7 after tumor challenge (28 days after the first immunization) and evaluated for HER-2-neu-specific antibody by ELISA (Figure 3). Interestingly, J-HER-vaccinated and TUBO-challenged mice had lower antibody titers to HER-2-neu than unvaccinated and TUBO-challenged mice. This is consistent with the lack or limited antibody generation by J-ICBL-based LEAPS vaccines [11, 14].

### 3.2. J-HER Immunization to Treat Established Tumor Progression

Mice were treated with the J-HER vaccine one week after initiation of tumor development by subcutaneous injection of TUBO cells into two sites (2.5 × 10^5^ cells per site). Vaccine treatments were repeated every two weeks after the initial treatment.

**Figure 4:**
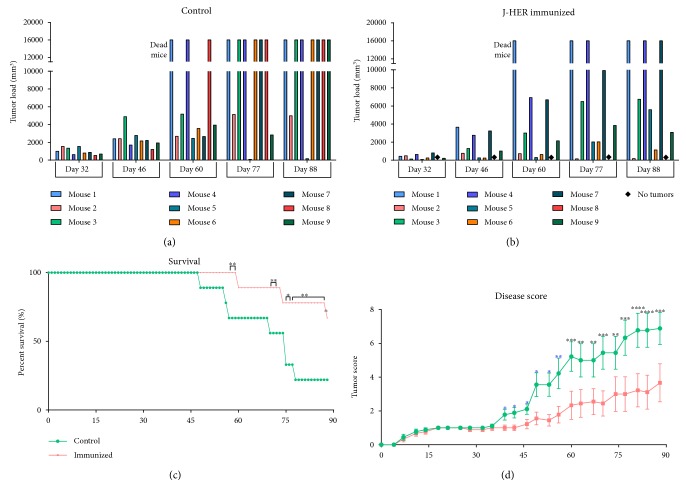
J-HER treatment of TUBO cell-challenged mice. Mice were challenged with 2.5 × 10 [7] TUBO cells in each of two sites and then immunized with HBSS in Montanide ISA-51 adjuvant (control *n* = 9) or J-HER in adjuvant (vaccine *n* = 9) one week later and at two-week intervals thereafter. Data is presented with respect to day after tumor challenge. (a) The average tumor load by volume for untreated mice. Mice that died during the trial are indicated by values of 16,000 and mice with no tumors are indicated by a diamond. (b) The average tumor load by volume for J-HER-treated mice, depiction as for panel (a). (c) Kaplan-Meier plot of survival on days shown after tumor challenge. (d) The disease score (calculated as defined in the Methods). (^∗^*p* < 0.05; ^∗∗^*p* < 0.01; ^∗∗∗^*p* < 0.001; ^∗∗∗∗^*p* < 0.0001.)

Unlike the untreated mice, J-HER-treated mice appeared otherwise healthy and tumor morbidity and mortality were significantly reduced (Figure 4). Mice treated with J-HER showed a lag in the development of tumors compared to untreated mice but still developed tumors (Figures 4(a) and 4(b)). The Kaplan-Meier depiction of survival clearly shows the benefits of J-HER treatment (Figure 4(c)). Mortality was delayed and more mice survived compared to untreated mice.

Since tumor load was only one indicator of the differences between untreated and J-HER-treated tumor-bearing animals, an alternative means of evaluation was developed to generate a total disease score of up to ten points based on the following: tumor load (0–3), presence of skin necrosis at the site of the tumor (+1), ascites (+1), general unkempt/unhealthy appearance (+1), and death (+4) (Figure 4(d)). The disease score at death was carried through for all subsequent days and calculations. Using this disease score, deviation of the outcomes became apparent 35 days after TUBO cell challenge coincident with the second immunization. The higher scores of the untreated mice represented the unkempt/unhealthy appearance of the mice (poor hygiene, lethargy, coat matting, ulceration, and wasting) and the greater number of deaths of untreated mice.

**Figure 5:**
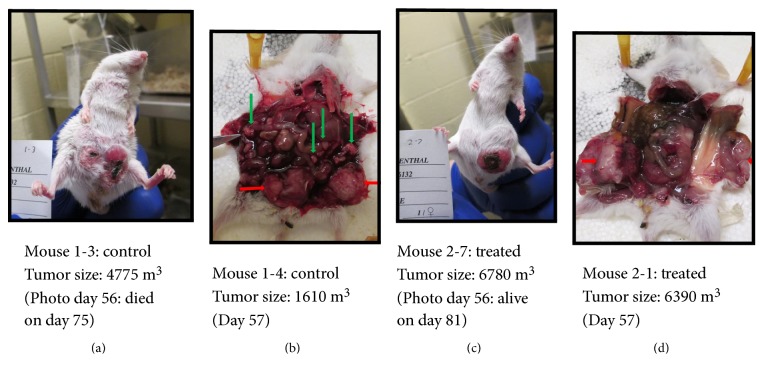
Appearance of representative mice from the J-HER treatment trial described in Figure 4. TUBO cell tumor development is shown for different control and J-HER-immunized mice. (a) Control, day 56, tumor size: 4775 m^3^, died on day 75. (b) Control, day 57, tumor size: 1610 m^3^. (c) Treated, day 56, tumor size: 6780 m^3^, survived the trial. (d) Treated, day 57, tumor size: 6390 m^3^. Red arrows indicate tumors at the site of implantation. Green arrows represent nodular tumors that spread within the abdomen.

The difference in mouse health can be seen in the pictures taken at necropsy on day 57 (Figure 5). Whereas untreated mice showed multiple tumor nodules spread throughout the abdomen, J-HER-treated mice showed a single tumor or limited tumor growth at the sites of the initial TUBO cell injection. The tumors did neither invade the abdomen nor spread and did not break through the skin and fur, as occurred for the untreated mice. This clearly shows that the J-HER treatment prevented the morbidity, mortality, and spread of tumors that occurred in the untreated mice.

## 4. Discussion

This pilot project demonstrated that the immune response elicited by J-HER was sufficient to prevent the initial development of tumor outgrowth in the TUBO breast cancer tumor model when administered prior to challenge. Presentation of J-LEAPS vaccines in an oil in water emulsion provides a reservoir for slow release is important for efficacy [14, 15] and why J-HER was administered in Montanide ISA-51. Others showed that strong adjuvants, such as TLR ligands, must be added to an oil in water emulsion (incomplete Freund's adjuvant) in order to elicit protection by the HER epitope [24], but this was not necessary with the J-HER vaccine.

The J-HER treatment limited tumor spread and the associated morbidity of disease progression but was not capable of eliminating the tumors that were initiated and then grew during the time period prior to immunization (one week) and required to elicit a sufficient immune response (~two weeks) to the J-HER treatment. Control of the spread of the tumors made a large difference in the health and survival of the J-HER-treated mice. It is likely that the response elicited by J-HER was capable of eliminating individual tumor cells when they arise, similar to the prevention trial, but was not able to enter or was insufficient to eliminate an established tumor.

Despite elicitation of protection in the prevention model and unlike most vaccines, the antibody response to the HER-2/neu protein in J-HER-immunized mice was less than that in unvaccinated mice. This suggests that antibody is not the primary means of prevention elicited by the J-HER vaccine. This is consistent with other J-LEAPS vaccines that elicit protection from disease and death upon HSV-1 challenge without detectable antibody production [9, 11, 14, 15].

Incorporating the HER-2/neu_66–74_ (HER) peptide into a J-LEAPS vaccine was intended to enhance the immunogenicity of the minimal MHC I epitope for CD8 T cells and direct the nature of subsequent immune responses towards Th1/Tc1. The HER peptide has limited immunogenicity due to its small size, as observed by others when administered in incomplete Freund's adjuvant [24]. The J-HER vaccine was designed based on previous findings for anti-HSV-1 vaccines [9, 14, 15]. Incorporation of three different CTL epitopes from HSV-1 into J-LEAPS vaccines elicited antigen-specific Th1/Tc1-related immune responses that were sufficient to provide protection in models with herpes simplex virus 1 (HSV-1) lethal challenge whereas the unconjugated peptides did not elicit measureable responses and had no protective activity. Incorporation of these epitopes into a similar-sized G- or derG-LEAPS vaccine failed to yield protective immunogens. The requirement for a CD8 T cell epitope in the J-LEAPS vaccine was indicated in studies with influenza vaccines in which attachment of the J-ICBL to CD8 T cell epitopes generated protective immunogens but attachment to CD4 T cell/B cell epitopes did not [16]. As for the anti-HSV-1 and anti-influenza vaccines, an effective vaccine was generated by covalent linkage of an appropriate CD8 T cell epitope (HER) from HER-2/neu to the J-ICBL.

As for the JgD (HSV) and JH (HIV) vaccines, immunization with J-HER is expected to promote the maturation of dendritic cell precursors and elicit production of IL12 and IFN*γ* [9, 14, 15]. Immunization of mice with the JgD anti-HSV vaccine without infection elicited IL12p70, IL17, and IFN*γ* serum responses in mice. Both the JgD vaccine and JH vaccines promoted maturation of murine bone marrow and human monocyte precursors to become IL12-producing dendritic cells [14, 15], ex vivo. The human JgD- and JH-induced DC1s elicited IFN*γ* production in mixed lymphocyte reactions [15], and murine JgD and JH DC1s elicited IFN*γ* production from T cells ex vivo. Adoptive transfer of the HSV-1-specific JgD-induced DC1s but not the HIV-specific JH-induced DC1s was sufficient to elicit antigen-specific protection from lethal HSV-1 challenge [14]. A J-HER-induced DC1 would also be expected to promote the development of Th1/Tc1 cells, and CTLs with greater sensitivity for MHC I presented tumor antigens [1–6].

The ability to limit potential metastatic spread or residual breast cancer disease by this type of vaccine response would be beneficial as a neoadjuvant therapy after surgical removal of the bulk tumors. Using similar logic, Koski, Czerniecki, and their collaborators demonstrated that HER-2/neu-loaded DC1 cells injected into the draining lymph node were effective as neoadjuvant therapy for breast cancer [7, 27]. Similarly, the J-HER peptide vaccine would be expected to promote maturation of DC1s, appropriate activation of CD8 T cells, and a systemic response that could attack individual metastatic tumor cells and prevent spread to lymph nodes.

With the demonstration that a J-HER immunization is effective for prevention and treatment in this proof of principle with the TUBO-challenge model, similar antitumor vaccines using alternative mouse or human CD8 T cell epitopes can be conceived. T cell-activating peptide vaccines can be customized for the tumor and the type of animal or for different individuals. Initial vaccine development would target antigens for the more common MHC molecules, such as HLA A2 [28]. The sequence of the relevant tumor antigen peptide can be predicted in silico or obtained from sequences reported in the literature. In addition to single-epitope vaccines, like J-HER, mixtures of J-LEAPS vaccines containing multiple epitopes from the same protein or different proteins can provide broader immune system coverage and minimize the chance of mutational escape by the tumor from immune surveillance. The CD8 T cell epitope targets can be optimized for the antitumor responses while excluding epitope targets associated with tolerance or autoimmune conditions. As peptides, single peptide or mixtures of antitumor J-LEAPS vaccines can be customized and synthesized for humans of different MHC types.

## Conflicts of Interest

K. S. Rosenthal is the coinventor on several patents on LEAPS technology but independent of CEL-SCI. Research on this project was performed independent of CEL-SCI. D. H. Zimmerman is an employee, officer, and stockholder of CEL-SCI Corp. and inventor and coinventor on multiple patents on LEAPS technology. The authors have no other relevant affiliations or financial involvement with any organization or entity with a financial interest in or financial conflict with the subject matter or materials discussed in the manuscript apart from those disclosed.
